# COMMUNITY COLLABORATOR AND OLDER ADULT EXPERIENCES WITH INTERVENTIONS DELIVERED BY OLDER ADULTS

**DOI:** 10.1093/geroni/igad104.1341

**Published:** 2023-12-21

**Authors:** Nicole Crawford, Amber Gum, Lawrence Schonfeld

## Abstract

This symposium will include researchers, collaborators from healthcare and social service organizations, and older adults serving as volunteer coaches or peer educators; speakers will describe their experiences conducting two randomized controlled trials involving behavioral interventions for older adults delivered by older adults. The first trial is a NIMH-funded three-site RCT for community-dwelling older adults with symptoms of depression, in which participants receive behavioral activation delivered by a masters-level clinician or a streamlined version (“Do More, Feel Better”) delivered by an older adult volunteer. This trial involves close collaboration with senior centers and an Area Agency on Aging (AAA). The second trial is a PCORI-funded RCT to reduce health disparities for Black/African-American and Hispanic/Latino older adults after medical hospitalization, in which participants receive the evidence-based Care Transitions Intervention (CTI), CTI plus a peer educator intervention, or usual care. This trial involves close collaboration with three regional hospital systems and an AAA. First, research investigators will describe the background, rationale, and methodology of each trial. Second, two representatives from the collaborating organizations (one from each trial) will present about their experiences collaborating on these trials – benefits of collaborating, challenges and strategies for overcoming challenges, and recommendations for other researchers engaged in similar community-based research. Third, two representatives from the older adult volunteers and peer educators (one from each trial) will present about their experiences collaborating on these trials – benefits of collaborating, challenges and strategies for overcoming challenges, and recommendations for other researchers engaged in similar community-based research.

